# Proximal Humerus Fracture: An Evaluation of the Readability and Value of Web-Based Knowledge

**DOI:** 10.7759/cureus.27957

**Published:** 2022-08-13

**Authors:** Mohamed Elshohna, Yasir Hidayat, Ahmed Karkuri

**Affiliations:** 1 Orthopaedics and Traumatology, University Hospital Limerick, Limerick, IRL; 2 Orthopaedics and Trauma, University Hospital Limerick, Limerick , IRL; 3 Orthopaedic Surgery, University Hospital Limerick, Limerick, IRL

**Keywords:** ireland, fracture, websites, humerus, shoulder

## Abstract

Background

In today’s scientifically developed world, the majority of patients use different websites to explore sophisticated and varied health knowledge. Consequently, healthcare specialists remain concerned that patients may be betrayed. Currently, there is a scarcity of information on the importance and legibility of online health data on proximal humerus fractures. This study aimed to assess the readability and value of existing web-based evidence regarding fractures of the proximal humerus.

Methodology

A search of three keywords, namely, broken shoulder, proximal humerus fracture, and broken humerus, was performed using the top three internet search engines. The first five pages of every search browser were analyzed. After discarding duplicate websites, 80 websites were found to be suitable for the analysis. Website quality was scored using the Journal of the American Medical Association (JAMA) benchmark criteria and the DISCERN criteria. The presence or absence of the Health on the Net Foundation Code of Conduct (HON code) certification and author characteristics were noted. The degree of readability was measured using six unique parameters, namely, the Automated Readability Index, Flesch Reading Ease Score, SMOG Index, Coleman-Liau index, Flesch-Kincaid Grade Level, and Gunning-Fog Index.

Results

In total, 80 specific websites were fit for evaluation and analysis. On the DISCERN tool, six (7.5%) websites revealed a high score. Only 20 websites fulfilled all four JAMA benchmark criteria. Of the total 80, only 17 were HON code-certified websites. Readability was variable but the majority was at the college level.

Conclusions

The most important result of this study is the low value, readability, and clarity of online testimony regarding proximal humerus fractures.

## Introduction

The Internet is an important international electronic network for individuals seeking information pertaining to almost any topic, including health information and healthcare services [1]. Studies have reported that the majority of orthopaedic outpatients depend largely on various websites for gaining important medical material [2]. Moreover, the majority of these patients do not disclose this knowledge to their treating physicians to enable mutual decision-making [3].

Patients are turning to peers on the Internet for information on everyday health issues. However, the mainstream continues to rely on health professionals for diagnoses, drug information, and recommendations about healthcare professionals or facilities [4].

The ability to find, read, comprehend, and apply medical information to make appropriate medical decisions and follow instructions for treatment in the public sector can be defined as health literacy, which is a major indicator of health status and effect. Low health literacy has been proven to be correlated with low compliance and longer hospital stay [5].

It is clear that proximal humerus fractures are widespread and create significant medical questions as they are the seventh most common fractures in the adult population [4]. The incidence of these fractures varies from 4% to 10% of all bony injuries, consistent with numerous studies performed in separate populations [6]. The management of these injuries is at times controversial, and several fracture patterns may be technically challenging [6]. The incidence of these fractures appears to be rising along with the increase in the elderly populations [7].

It is clear that medical knowledge on various websites about the different management methods for these fractures is rich; however, it is associated with an absence of clarity and poor clear information on fractures of the proximal humerus [3]. According to the medical literature, health material should be at a level that is understandable to at least sixth-grade students [8].

The necessity to supply the population with accurate and readable medical information was the motivation to appraise the online information regarding proximal humerus fractures.

## Materials and methods

**Figure 1:**
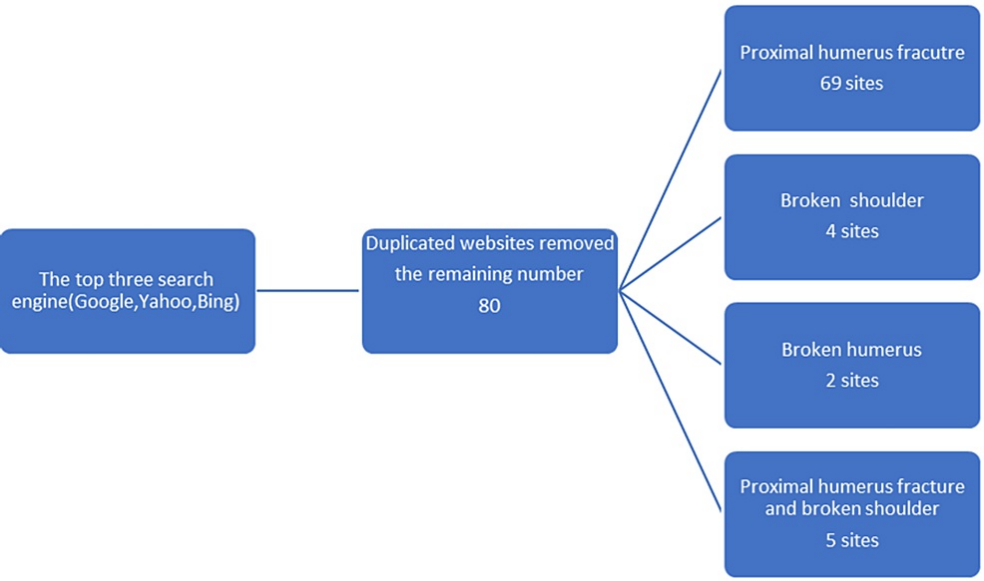
Internet search flowchart. The figure demonstrates the process of website collection and analysis.

We conducted this research using three separate keywords, namely, broken shoulder, proximal humerus fracture, and broken humerus, utilizing the leading internet browsers (Google, Bing, and Yahoo) on March 2, 2022. The three search engines are responsible for more than 99% of the market share in March according to www.StatCounter.com [9]. An analysis of the first five pages from each browser was done and collected in an excel sheet. After eliminating duplicates and similar websites, 80 websites were included in the analysis (Figure 1). A directory of all internet sites is available in Appendix 1.

**Figure 2:**
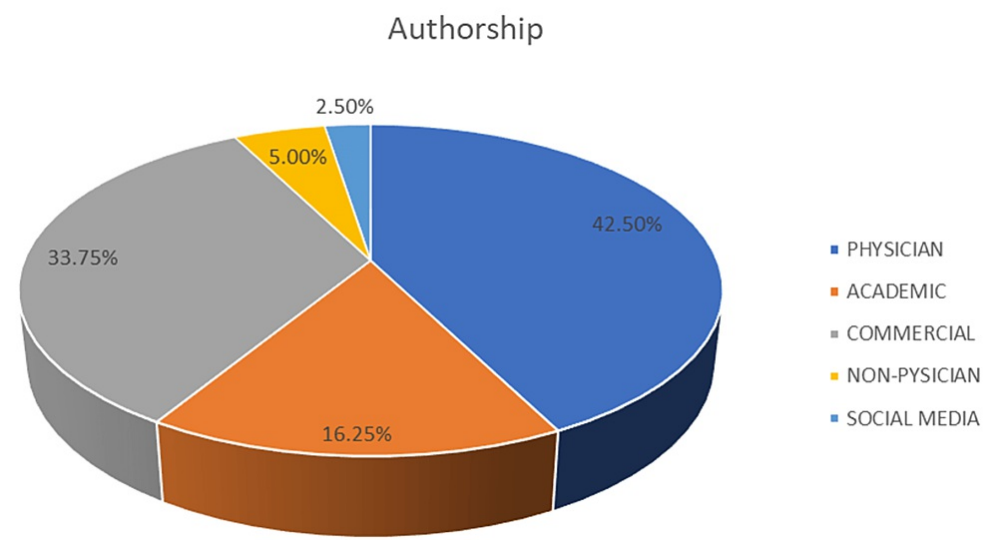
Authorship classification. The pie chart shows four different categories regarding authorship, namely, academic, physician, non-physician, commercial, and social media.

Four different categories regarding the creators and owners of the websites were used, namely, academic, physician, non-physician, commercial, and social media (Figure 2).

The evaluation of quality was accomplished by two separate authors using the well-known three validated tools, namely, Health on the Net Foundation Code of Conduct (HON Code), DISCERN, and the Journal of the American Medical Association (JAMA) benchmark criteria, within three weeks of the original search. DISCERN is a website designed to support the researchers of medical information in verifying the importance of authored knowledge regarding management plans [10]. This instrument comprises 16 questions, each rated on a five-point scale, where a rating of 1 means the criterion was not satisfied, 2-4 means the criterion is partially satisfied, and 5 means the complete fulfilment of the criterion. This is a vital standard that plays a significant role in assessing the accuracy of the information on treatment choices [11].

The JAMA benchmark assesses the next four essential values, namely, authorship (affiliations, contributors, authors, and credentials), attribution (sources used for the content, copyright information, and references), disclosures (potential conflicts of interests, advertising, commercial funding, and sponsorship), and, finally, the currency (dates of posted and updated information) [12].

The HON code quality and the certification process have been developed by the Health on the Net Foundation to improve the transparency of the health and medical information found on the Internet [13].

www.webfx.com is an online application which was used to calculate the readability score; however, this application was not able to analyse three websites. Accordingly, another application was used, www.seoreviewtools.com. The calculated score reflects the grade level needed to comprehend a passage of text. The grade level of the writing needs should accurately reflect the grade level of the target audience [10].

## Results

Quality analysis

The mean DISCERN score was 35.25 (16-75). According to the DISCERN tool, only six (7.5%) websites showed a high-level score, while 69 (86.25%) websites had low-down scores from fair, poor, and very poor results (Table 2).

**Table 1:** DISCERN score.

DISCERN score	Number of websites
Excellent is denoted by scores of 63 to 75 points	6
Good is denoted by scores of 51 to 62 points	5
Fair is denoted by scores of 39 to 50 points	13
Poor is denoted by scores of 27 to 38 points	33
Very poor is denoted by scores of 16 to 26 points	23

**Figure 3:**
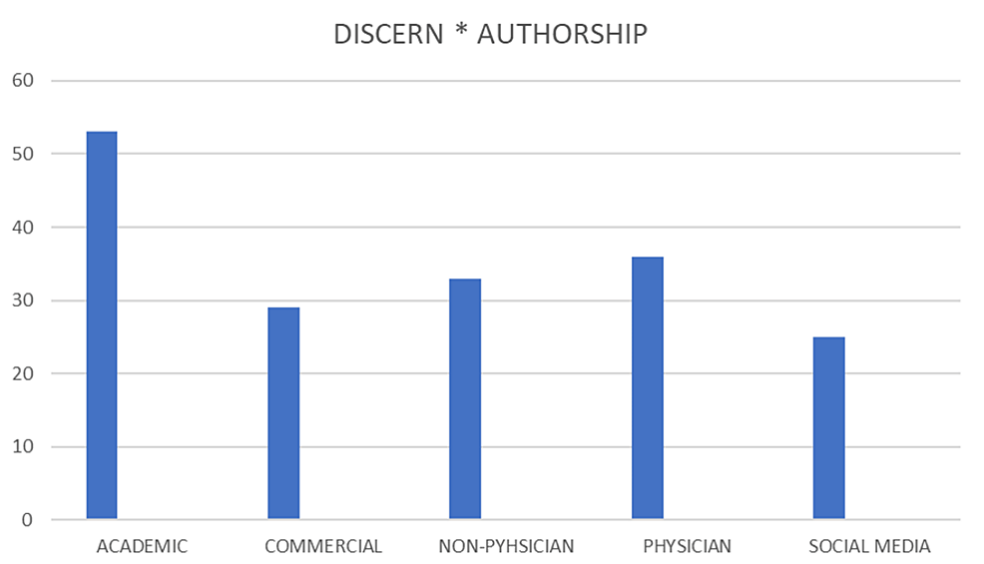
DISCERN data. The figure illustrates the relationship between the authorship categories and the DISCERN score.

The websites with academic authorship had a high DISCERN score when linked with the others. The sites with an HON code certificate also gained high scores on the DISCERN tool. Moreover, commercial and social media websites scored low with the DISCERN tool (Figure 3).

**Figure 4:**
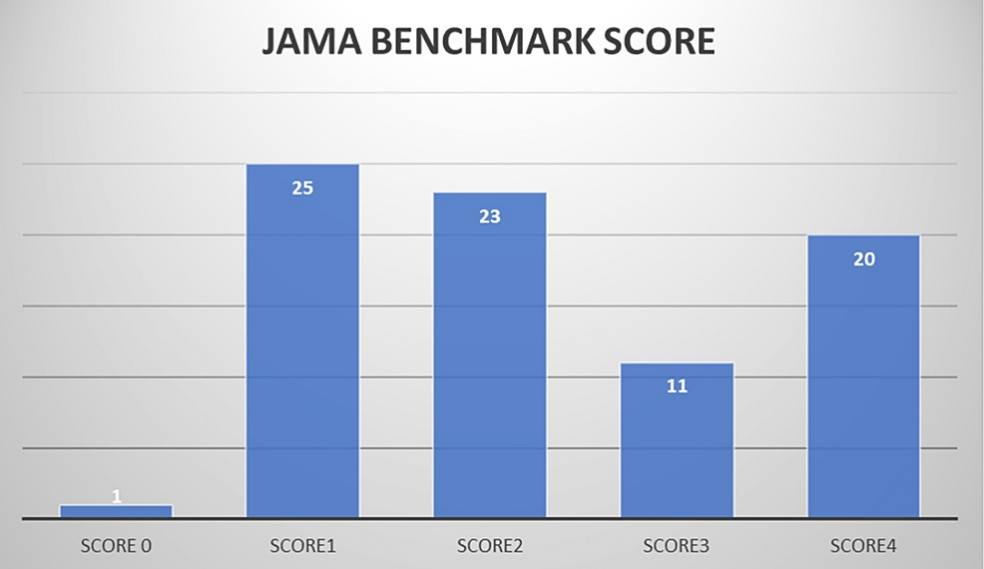
JAMA benchmark score. The figure demonstrates the JAMA Benchmark score from the lowest score of 0 to the highest score of 4.

Only 17 HON code-certified websites were observed out of a total of 80 (21.3 %). The mean JAMA score was 2.30(0-4). In total, 20 websites achieved all four JAMA benchmark criteria, although 25 scored only one (Figure 4).

Readability assessment

The mean Flesch Readability Ease Score (FRES) was 50.99 (-102.4-100.9), the mean Flesch-Kincaid grade level was 8.006 (1.0-29.0), and the mean Gunning Fog index was 8.23 (2.4-15.1). Only 20 sites had an FRES of >60 that matches with eighth-grade level. The mainstream of the 52/80 scored at the college level (FRES: 30-60); however, 8/80 had a score of less than 30, equivalent to increasing difficulty in comprehension and reading (Table 3).

**Table 2:** Readability scores. The table shows the calculations of six different scores used to assess the readability.

	Flesch Reading Ease Score	Flesch-Kincaid Grade Level	Gunning Fog Score	SMOG Index	Coleman-Liau index	Automated Readability Index
Mean	50.990	8.006	8.235	6.903	14.655	6.587
Median	50.050	8.100	8.600	6.900	14.900	7.000
Mode	46.0^a^	8.1	2.4^a^	7.7	15.2	7.4
Variance	618.733	12.864	8.408	2.814	22.290	15.904
Range	203.3	28.0	12.7	8.0	37.4	29.8
Minimum	-102.4	1.0	2.4	3.2	4.7	-1.9
Maximum	100.9	29.0	15.1	11.2	42.1	27.9
Sum	4079.2	616.5	634.1	531.5	1128.4	507.2

## Discussion

The Internet has grown to become an indispensable part of everyday life. Although it has improved everything around us and eased obtaining knowledge and working together for many people, there are issues regarding the reliability of the available data. This relates to various topics, including medical knowledge about fracture management. In this cyberspace age, people seek data on different websites generally without specialized assistance. Looking for medical information is the third most popular interest on the Internet [14].

In the past, few medical information sources were available to the public which were authored by health professionals. Because of recent advances in our society influenced by the availability of medical information on many websites and the need for patients to be well informed about their medical issues, it has become easier to get medical data through the Internet. The presence of dependable health information is valuable to sick people who have to make minor or major medical decisions as a well-informed patient can make more accurate medical decisions. Present reliable literature has shown that when a patient participates in decision-making, better individual values are obtained [15]. To date, no study has assessed the quality of medical information available to the general population on the websites regarding proximal humerus fractures. Considering the huge amount of knowledge present on the Internet, it can mislead the general population when it is of poor value. Consequently, numerous tools such as the JAMA benchmark, DISCERN, and the HONcode have been designed to scrutinize the material on various medical sites [16].

According to the results of this research, the hypothesis is rejected because the quality of Internet data on proximal humerus fractures is mainly poor but comprehensible. Erratically, of the 80 websites, only 17 were HON code certified. The HON code application allows web customers to scrutinize whether they can rely on the knowledge found. Fundamentally, it was established as a project to guarantee high-quality medical data. The HON code certification is asked for by various web publishers with a self-evaluation measure. Next, the HON code Review Committee supervises a systematic review and offers references. Depending on the agreement with these requests, the website is awarded the HON code certification, which is applicable for only one year [17].

In our study, the information accessible on the Internet is of low to moderate quality on medical issues. Inconsistencies in the authorship groupings on both the DISCERN score and JAMA benchmark criteria were noted; nevertheless, physician-registered and academic online sites offered better-quality information, followed by other groups. It was disturbing that the mean JAMA grade was within the standard (2.30) similar to systemic reviews about orthopaedic sports medicine (2.00), indicating poor information quality [18]. Apart from the JAMA tool focusing on numerous characteristics of the Internet other than the content of which users might not be aware, knowledge about these qualities is good from the point of view of the scientific community. The JAMA benchmark is a highly efficient tool for medical value assessment, allowing researchers to quickly discredit the medical websites that do not have the highly necessary elements of data clarity and reliability [12]. The extremely significant inadequacy of the material quality is only 20 websites achieved all four JAMA benchmark standards while a substantial number of the websites (25) scored only one.

The DISCERN application scrutinizes the value of medical websites using 15 separate questions involving the various characteristics of the included knowledge and one general summarizing question [11]. Based on the DISCERN tool, only 7.5% of the websites had a high score, while 69 (86.25%) websites had low scores ranging from fair, poor, and very poor, revealing the low quality of information. Most of the drawbacks regarding the DISCERN tool originated from scarcity of or insufficient material on the different methods of treating proximal humerus fracture and the importance of professional (mechanical/surgical) therapy; consequently, commercial and social media websites scored low on this tool [11].

The assessment of the websites showed simple and readable text, which is understandable by the general public. Furthermore, there were significant associations between the number of sentences with FKG and FRES grades. Despite the fact that medical information is best written at the student degree at fifth and sixth grades, the calculated information repeatedly showed that data on proximal humerus fractures on the Internet is written at approximately the 10th to 12th grades [10]. In this research, the mean FRES, FKG, and GFI grades were 50.99, 8.006, and 8.23, respectively, which are greater than the mentioned sixth-grade comprehension level, as supported by the American Medical Association (AMA) [15]. Only 25% of websites had an FRES score of >60 at par with the eighth-grade level. The website https://www.youtube.com/watch?v=1HEM4c76rQ4 had the most difficult content as it has average reading ease of about -102.4 of 100 which is very complicated. In contrast, the website https://www.northumbria.nhs.uk/sites/default/files/images/PIN679%5B1%5D_0.pdf had the highest score as it had average reading ease of about 100.9 of 100. It should be easily understood by seven to eight-year-olds, mainly due to the satisfactory number of short sentences, even though the text was long [10]. One of the explanations for getting great readability grades is that 58.75 % of the websites are either academic or physician collected, as stated previously. Because the present research is the first to evaluate the readability of written content on proximal humerus fracture, it is challenging to compare the outcomes with the available literature [16].

This study’s shortcomings are that we were not able to fit sanctions to particular parts of the patients. Another possible disadvantage of this research is that the reliability of demonstrations of HON code certificates itself has been questioned [20]. Additionally, this research could not clarify the misleading utilization of the HON code certificate. Even though we examined three keywords across three popular search engines, the result was restricted to the first five pages on each engine. This study has confirmed that online websites are an encouraging tool for public health and healthcare and a potentially effective platform for health communication and education [19]. Future studies should concentrate on the approach to the Internet, as well as its effects on search activities and search period difficulty within various sections of the population.

## Conclusions

A crucial finding of this study was the low-down value and comprehensibility of online evidence regarding proximal humerus fractures. A shortage of easily comprehensible and available Internet sites was noticed, which can possibly affect patient outcomes.

## Appendices

**Table 3:** A directory of all Internet sites.

Websites
http://www.arcticorthopedics.com/proximal-humerus-fracture-broken-shoulder-2/
http://www.bonetalks.com/armproxhum
http://www.guildfordupperlimb.co.uk/shoulder/humerus-fracture-shoulder
http://www.inovamedicalgroup.org/upload/docs/Inova%20Medical%20Group/Ortho%20Trauma%20Program/Proximal%20Humerus%20Fracture.pdf
https://bmcmusculoskeletdisord.biomedcentral.com/articles/10.1186/s12891-019-2459-6
https://bostonshoulderinstitute.com/patient-resources/modules/proximal-humerus-fracture/
https://centralcoastortho.com/patient-education/proximal-humerus-fracture-broken-shoulder/
https://melbournearmclinic.com.au/proximal-humerus-fracture/
https://my.clevelandclinic.org/health/diseases/17470-shoulder-fractures
https://orthofixar.com/trauma/proximal-humerus-fracture/
https://orthoinfo.aaos.org/en/diseases--conditions/shoulder-trauma-fractures-and-dislocations/
https://orthosports.com.au/shoulder/proximal-humeral-fractures/
https://ota.org/for-patients/find-info-body-part/3831
https://patient.info/bones-joints-muscles/broken-upper-arm
https://proximalhumerusfracture.blogspot.com/
https://pubmed.ncbi.nlm.nih.gov/16156469/
https://pubmed.ncbi.nlm.nih.gov/31948835/
http://rebalancemd.com/wp-content/uploads/2017/08/Proximal_Humerus_Fracture_Recovery_Guide.pdf
https://radiologykey.com/proximal-humerus-fractures-and-shoulder-dislocations/
https://radiopaedia.org/articles/proximal-humeral-fracture-1
https://rileywilliamsmd.com/shoulder-fracture-proximal-humerus-broken-scapula-orthopedic-shoulder-specialist-manhattan-new-york-city-ny/
https://smortho.ca/patient/proxhumerus/
https://tcomn.com/wp-content/uploads/2015/04/The-Proximal-Humerus-Fracture-Book.pdf
https://thecoreinstitute.com/wp-content/themes/the-core/documents/patient-education/Proximal-Humerus-Fractures_Patient_Education_PE_SH_%207-16-2019.pdf
https://www.armdocs.com/condition/proximal-humerus-fracture
https://www.chelwest.nhs.uk/your-visit/patient-leaflets/surgery-services/shoulder-injury-fractured-proximal-humerus
https://www.childrenshospital.org/-/media/Conditions-and-Treatments/Conditions/Fractures/proximal-humeral-fracture-guide.ashx?la=en&hash=DDE41AACBD92CE0CC60C2DB08BC52E22D9745D79
https://www.choosept.com/guide/physical-therapy-guide-proximal-humerus-fractures
https://www.dd-clinic.com/spotlight/proximal-humerus-fracture-broken-shoulder/
https://www.drgordongroh.com/orthopaedic-injuries-treatment/shoulder/shoulder-fractures-proximal-humerus/
https://www.drugs.com/cg/proximal-humerus-fracture.html
https://www.esht.nhs.uk/leaflet/proximal-humerus-fracture/
https://www.esht.nhs.uk/wp-content/uploads/2017/06/0628.pdf
https://www.fortiusclinic.com/conditions/shoulder/proximal-humerus-upper-arm
https://www.fracturecare.co.uk/care-plans/shoulder/proximal-humerus-fracture/
https://www.healthline.com/health/humerus-fracture
https://www.hey.nhs.uk/patient-leaflet/fracture-humerus-advice-sheet/
https://www.hopkinsmedicine.org/health/conditions-and-diseases/humerus-fracture-upper-arm-fracture
https://www.iow.nhs.uk/Downloads/Patient%20Information%20Leaflets/fracture%20clinic%20-%20care%20of%20your%20humera_upper%20arm_fracture.pdf
https://www.lancasterortho.com/conditions/shoulder/proximal-humeral-shoulder-fracture.html
https://www.livestrong.com/article/160243-home-rehabilitation-exercises-for-a-broken-upper-humerus/
https://www.mdpi.com/2075-1729/12/2/311/html
https://www.medicinenet.com/how_painful_is_a_broken_humerus/article.htm
https://www.medstarhealth.org/services/proximal-humerus-fracture-shoulder-fracture
https://www.mgo.md/patient-resources/education/proximal-humerus-fracture
https://www.ncbi.nlm.nih.gov/books/NBK470346/
https://www.ncbi.nlm.nih.gov/pmc/articles/PMC3535090/
https://www.ncbi.nlm.nih.gov/pmc/articles/PMC5788098/
https://www.northumbria.nhs.uk/sites/default/files/images/PIN679%5B1%5D_0.pdf
https://www.orthobullets.com/pediatrics/4004/proximal-humerus-fracture--pediatric
https://www.orthobullets.com/trauma/1015/proximal-humerus-fractures
https://www.orthopaedicsone.com/display/MSKMed/Proximal+Humerus+(shoulder)+fractures
https://www.phoenixrehabgroup.com/proximal-humerus-fracture.html
https://www.rch.org.au/clinicalguide/guideline_index/fractures/Proximal_humeral_fractures_Emergency_Department/
https://www.renoortho.com/specialties/center-for-fracture-trauma/proximal-humerus-fracture/
https://www.researchgate.net/publication/358888595_The_reliability_of_the_Neer_classification_for_proximal_humerus_fractures_A_survey_of_orthopedic_shoulder_surgeons
https://www.resurgens.com/shoulder/conditions/proximal-humerus-fracture
https://www.ruh.nhs.uk/patients/patient_information/ORT039_Advice_after_a_shoulder_fracture.pdf
https://www.saintlukeskc.org/health-library/understanding-humerus-fracture
https://www.samtaylormd.com/conditions/proximal-humerus-fracture/
https://www.shoulderdoc.co.uk/article/735
https://www.shoulderdoc.co.uk/news/view/169
https://www.shoulderdoc.co.uk/news/view/934
https://www.shoulderelbowclinic.co.uk/proximal-humeral-fractures
https://www.shoulder-pain-explained.com/humerus-fracture.html
https://www.shoulder-pain-explained.com/proximal-humerus-fracture.html
https://www.shouldersurgeon.com/proximal-humerus.html
https://www.shouldersurgery.com.au/shoulder-fractures.html
https://www.sirona-cic.org.uk/advice-information/leaflet-library/musculoskeletal-msk-services/leaflet-proximal-humerus-fracture-exercises/
https://www.sports-health.com/sports-injuries/shoulder-injuries/3-types-shoulder-fractures
https://www.sports-health.com/sports-injuries/shoulder-injuries/proximal-humerus-fractures-shoulder
https://www.sports-health.com/sports-injuries/shoulder-injuries/treating-proximal-humerus-fracture
https://www.stevenchudikmd.com/knee-surgeon-chicago-illinois/injuries-conditions/proximal-humerus-fracture/
https://www.stgeorges.nhs.uk/wp-content/uploads/2020/07/THE_PHF_01.pdf
https://www.verywellhealth.com/humerus-fracture-2549285
https://www.verywellhealth.com/physical-therapy-after-a-proximal-humeral-fracture-2696019
https://www.verywellhealth.com/proximal-humerus-fracture-2548596
https://www.windsorupperlimb.com/conditions/shoulder-conditions/common-shoulder-fractures/proximal-humerus-fractures
https://www.youtube.com/watch?v=1HEM4c76rQ4
https://yorkshireshoulder.com/proximal-humerus-fractures/
