# Correction: Proximal Humerus Fracture: An Evaluation of the Readability and Value of Web-Based Knowledge

**DOI:** 10.7759/cureus.c72

**Published:** 2022-08-23

**Authors:** Mohamed Elshohna, Yasir Hidayat, Ahmed Karkuri

**Affiliations:** 1 Orthopaedics and Traumatology, University Hospital Limerick, Limerick, IRL; 2 Orthopaedics and Trauma, University Hospital Limerick, Limerick , IRL; 3 Orthopaedic Surgery, University Hospital Limerick, Limerick, IRL

This article was submitted and published without including the second and third authors due to an error by the submitting author. The authors deeply regret this error. The article has now been corrected to include Yasir Hidayat and Ahmed Karkuri.

